# Merestinib monotherapy or in combination for japanese patients with advanced and/or metastatic cancer: A phase 1 study

**DOI:** 10.1002/cam4.4110

**Published:** 2021-09-09

**Authors:** Toshihiko Doi, Noboru Yamamoto, Yoichi Naito, Yasutoshi Kuboki, Takafumi Koyama, Yongzhe Piao, Naoto Tsujimoto, Hiroya Asou, Koichi Inoue, Shunsuke Kondo

**Affiliations:** ^1^ National Cancer Center Hospital East Chiba Japan; ^2^ Department of Experimental Therapeutics National Cancer Center Hospital Tokyo Japan; ^3^ Eli Lilly Japan K.K Kobe Japan

**Keywords:** biliary tract neoplasms, carcinoma, metastasis, phosphotransferases, toxicity

## Abstract

This phase 1, multi‐center, nonrandomized, open‐label, dose‐escalation study consisted of Part A wherein merestinib 80 or 120 mg (40‐mg tablets) was administered orally QD during a 28‐day cycle to patients diagnosed with solid tumors and Part B wherein merestinib 80 mg (40‐mg tablets) was administered orally QD, and cisplatin 25 mg/m^2^ + gemcitabine 1000 mg/m^2^ administered IV on Day 1 and Day 8 of a 21‐day cycle (for a maximum of eight cycles) to patients diagnosed with biliary tract carcinoma (BTC). Nineteen patients were screened and 18 patients were (Part A, *n* = 10; Part B, *n* = 8) enrolled in the trial and received treatment. All patients in Parts A and B were from Japan and were within an age range of 43–73 years, with an ECOG PS of 0.1. No dose‐limiting toxicity or deaths were experienced in the study. Dose‐limiting toxicity equivalent toxicity of Grade 4 platelet count decreased (*n* = 1) and was observed in Part B. In Part A, treatment‐related Grade ≥3 TEAEs were reported in one patient (PT: ALT increased and AST increased), while in Part B, five patients reported treatment‐related Grade ≥3 TEAEs with four of the five patients reporting an event of neutrophil count decreased. No complete response was reported in either Part. One patient in Part B reported partial response while four patients in each part reported stable disease. Merestinib monotherapy was concluded to be tolerable in Japanese patients, and its combination with cisplatin and gemcitabine is a tolerable regimen for Japanese patients with BTC.

Trial registration: NCT03027284 (ClinicalTrials.gov) registered on 23 January 2017.

## Graphical abstract

The article describes the findings of Study I3O‐JE‐JSBG. The results of this phase 1, multicenter, nonrandomized, open‐label, dose‐escalation study showed that merestinib monotherapy is tolerable in Japanese patients, and its combination with cisplatin and gemcitabine is a tolerable regimen for Japanese patients with biliary tract cancer.

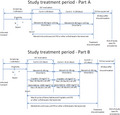

## 1 INTRODUCTION

The tyrosine kinase receptor MET (c‐Met), also known as hepatocyte growth factor receptor (HGFR), the product of the *MET* gene, has many downstream effects including cell survival regulation and migration of epithelial and myogenic precursor cells.^1^ Aberrant MET signaling, which may be an outcome of genetic lesions, transcriptional upregulation, or ligand‐dependent autocrine or paracrine mechanisms, plays a crucial role in tumorigenesis and angiogenesis.^2^ Tumor biopsies of most solid tumors show an increased expression of MET and its ligand HGF.^3^, ^4^, ^5^ In addition, MET dysregulation is associated with the development of resistance to therapies targeting epidermal growth factor receptors (EGFRs), such as erlotinib and gefitinib.^6^, ^7^

MET expression is also a common feature of biliary tract carcinoma (BTC) which includes a range of adenocarcinomas including cancer of the gallbladder, intra‐ and extra‐hepatic biliary ducts, and ampulla of Vater.^8^, ^9^ Interestingly, globally, it has been observed that the incidence of cholangiocarcinoma (CCA) is substantially higher in parts of the Eastern world compared with the West.^10^ In Japan, in particular, BTC is the seventh leading cause of cancer mortality,^11^ reporting very poor prognosis as the majority of patients present only at advanced stages with unresectable tumors.^12^, ^13^ A significant negative correlation has been established between high expression of c‐MET and overall and disease‐free survival in patients with intrahepatic CCA.^14^ Although the phase 3 randomized ABC‐02 trial (NCT00262769) established the combination of cisplatin and gemcitabine as a reference standard for the treatment of BTC, 50% of the patients succumb to the disease at 1 year thus demonstrating an immense unmet medical need.^15^

Merestinib (LY2801653) is a potent and selective type II c‐Met kinase inhibitor capable of inhibiting c‐Met activity both in vitro and in vivo. It has also been shown to be an inhibitor of several other oncokinases, including 13 MET mutations/variants.^16^ Merestinib has demonstrated single‐agent inhibitory activity against oncokinases like MET, AXL, ROS1, and NTRK.^17^

A previously conducted phase 1 study^18^ demonstrated that merestinib had a clinically acceptable safety profile and potential antitumor activity in patients with advanced cancer in the US warranting further clinical investigation in specific disease groups. Since it is imperative to optimize dose regimen and ensure safety and efficacy across ethnicities to account for differences in pharmacogenomics, pharmacoeconomics, social environment, regulatory pathways, and regional medical care,^19^ this study aimed to evaluate the tolerability, safety, and pharmacokinetics (PK) of merestinib monotherapy or in combination with gemcitabine and cisplatin agents in Japanese patients with advanced/metastatic cancer and BTC, respectively.

## 2 METHODS

### 2.1 Study design

**FIGURE 1:**
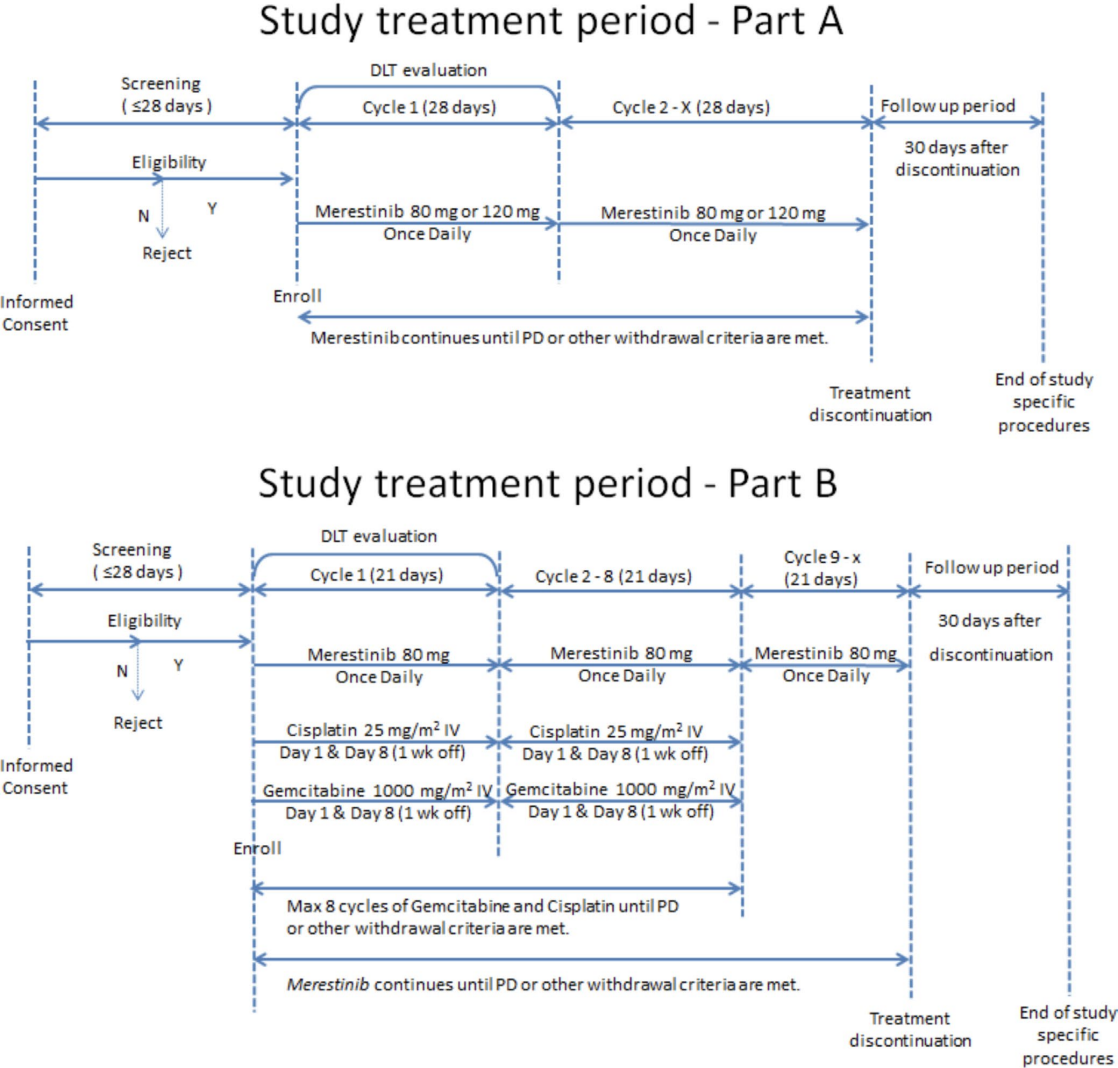
Study designs

Study JSBG was a multi‐center, open‐label, non‐randomized, Phase 1 study of merestinib. It consisted of a 3 + 3 dose‐escalation part (Part A) in Japanese patients with advanced and/or metastatic cancer (solid tumors or non‐Hodgkin’s lymphoma) and a combination part (Part B) in Japanese patients with unresectable, recurrent, or metastatic BTC. Figure 1 demonstrates the study design.

The primary objective of this study was to evaluate the tolerability of merestinib monotherapy or in combination with other gemcitabine and cisplatin in Japanese patients with advanced and/or metastatic cancer. The secondary objectives included evaluation of safety and toxicity profile, PK profile, and antitumor activity of merestinib in monotherapy or in combination with gemcitabine and cisplatin in the aforementioned patient pool.

The study protocol was approved by Institutional Review Boards prior to patient recruitment, and each patient provided written informed consent before enrollment. The study was conducted in accordance with consensus ethics principles derived from international ethics guidelines, including the Declaration of Helsinki and the Council for International Organizations of Medical Sciences International Ethical Guideline and the International Conference on Harmonization E6 Guidelines for Good Clinical Practice.

### 2.2 Patient population

Part A included Japanese patients, ≥20 years of age, diagnosed with advanced and/or metastatic cancer (solid tumors or non‐Hodgkin’s lymphoma); measurable or non‐measurable disease, as defined by RECIST Version 1.1. Part B included Japanese patients, ≥20 years of age, diagnosed with BTC that was unresectable, recurrent, or metastatic and for the treatment of which prior systemic front‐line therapy was not received; measurable disease, as defined by RECIST Version 1.1. Patients with serious preexisting medical conditions such as liver cirrhosis with a Child‐Pugh state of B or higher, symptomatic central nervous system malignancies or metastasis, history of cardiac conditions, active infections, active interstitial lung disease, or a secondary primary malignancy were excluded.

### 2.3 Study treatment

Part A consisted of two dose levels of merestinib: 80 mg (dose level 1) or 120 mg (dose level 2). Merestinib was supplied as 40‐mg tablets for oral administration and the assigned dose was administered QD, in a 28‐day cycle. The second dose level of 120 mg was administered if <33% of patients on dose level 1 (merestinib 80 mg), had dose‐limiting toxicity (DLT) in Cycle 1. If in Cycle 1, the frequency of DLT was the initial dose and reduced dose for dose level 1 were 80 and 40 mg/day, respectively, and the initial dose and reduced dose for dose level 2 were 120 mg/day (three tablets of 40 mg merestinib) and 80 mg/day (two tablets of 40 mg merestinib), respectively.

In Part B, treatment with merestinib (80 mg; two tablets of 40 mg merestinib) in combination with cisplatin and gemcitabine was initiated to run concurrent with the second dose level (120 mg) of Part A. The assigned dose of merestinib (80 mg) was administered orally QD in a 21‐day cycle. Cisplatin dose was 25 mg/m^2^ intravenous (IV) and the gemcitabine dose was 1000 mg/m^2^ IV, with each drug administered on Day 1 and Day 8 of repeating a 21‐day cycle (weekly with 2 weeks on and 1 week off). Treatment with cisplatin and/or gemcitabine could continue for a planned maximum of eight cycles, or until no further clinical benefit, excessive toxicity or evidence of disease progression if merestinib continued.

For both parts, the planned duration of treatment with merestinib was not fixed and patients remained on the study until progressive disease (PD), development of unacceptable toxicity, or fulfilment of one of the criteria for study discontinuation.

### 2.4 Safety

Safety and tolerability were assessed based on DLT analysis, and clinical and laboratory evaluations. The Common Terminology Criteria for Adverse Events version 4.0 were used for grade adverse events (AEs). Graded AEs were subsequently coded to Medical Dictionary for Regulatory Activities preferred terms. All patient deaths were also recorded.

In Part A, DLT was defined as an AE related to merestinib during Cycle 1 (28 days) while in Part B DLT was defined as an AE related to merestinib, and not a reasonably anticipated AE related to cisplatin and gemcitabine, during Cycle 1 (first 21 days). In addition, one of the following criteria had to be fulfilled: Grade ≥3 non‐hematological toxicity (except diarrhea, nausea, anorexia, vomiting, constipation, and fatigue; transient ≤7 days grade ≥3 elevations of ALT and/or AST; asymptomatic electrolyte disturbance that can be treated with oral substitution therapy); Grade 3 thrombocytopenia with grade ≥2 bleeding or thrombocytopenia which requires platelet transfusion; Grade 4 neutropenia of >7 days duration; any febrile neutropenia; anemia which requires a transfusion of packed red blood cells; Grade 4 thrombocytopenia (only for Part A).

The DLT evaluation period referred to the 28 days period of Cycle 1 for Part A, and the first 21 days of Cycle 1 for Part B while DLT evaluable population included all enrolled patients who took at least 80% of assigned dose in the DLT evaluation period for all study treatments or a patient who experienced a DLT in the DLT evaluation period.

Dose adjustments were carried out for merestinib, gemcitabine, and cisplatin based on DLT or DLT‐equivalent toxicity as defined in the study protocol. Adjustments for merestinib were carried out as dose omission and dose reduction steps, while they were carried out for gemcitabine as dose delay, dose reduction, and dose interrupted steps. Adjustments for cisplatin, on the other hand, were carried out as dose delay, and dose reduction steps.

### 2.5 Pharmacokinetics

Pharmacokinetic analyses were conducted on patients who had received at least one dose of study treatment and had sufficient samples collected throughout the first two cycles to allow the estimation of PK parameters.

Standard non‐compartmental methods of analysis using Phoenix WinNonlin® 8.1 (Certara, L.P.) were employed to determine the PK parameter estimates for merestinib and its pharmacologically active metabolites (LSN2800870 and LSN2887652) were calculated by standard non‐compartmental methods of analysis using Phoenix WinNonlin® 8.1 (Certara, L.P.). The primary parameters for analysis were maximum observed plasma concentration (*C*_max_), area under the plasma concentration–time curve from time zero to last measurable plasma concentration (AUC_0–tlast_), and area under the plasma concentration–time curve from time zero to infinity (AUC_0–∞_) of merestinib. Other non‐compartmental parameters, such as half‐life (*t*_1/2_), apparent systemic clearance (CL/F), and apparent volume of distribution (*V*_z_/*F*) were also assessed.

### 2.6 Antitumor activity

Based on the type of histology, tumor response was assessed and recorded using RECIST v1.1 for solid tumors. The following efficacy endpoints were used: objective response rate (ORR), disease control rate defined as a percentage of patients who are either (i) responders (exhibit CR or PR), or (ii) have stable disease (SD) for at least 6 weeks divided by the total number of patients to the corresponding part and/or cohort, best overall response, change in tumor size (solid tumors only), duration of response, and duration of SD.

### 2.7 Statistical analysis

Descriptive statistics (patient number, mean, median, SD, range, minimum, maximum for continuous variables, patient number, frequency, percentages, and standard errors for categorical variables) were used to summarize the data, and *p* values were not calculated all analyses were descriptive; no *p* values were calculated. For continuous variables, summary statistics included number of patients, mean, median, standard deviation, minimum, and maximum. Categorical endpoints were summarized using the number of patients, frequency, percentages, and standard errors. No imputation was made for missing data were not imputed.

## 3 RESULTS

### 3.1 Patient disposition and demographics

A total of 19 patients were screened and 18 patients were (Part A, *n* = 10; Part B, *n* = 8) enrolled in the trial and received treatment. Of the three patients who were assigned to Part A, dose level 1, all patients received at least one dose of study treatment. Of the eight patients who were assigned to Part A, dose level 2, seven patients received at least one dose of study treatment. Of the eight patients who were assigned to Part B, all patients received at least one dose of study treatment. All patients in Parts A and B were Asian and from Japan and were within an age range of 43–73 years, with an ECOG PS of 0 and 1. More than half of the patients in Part A (dose level 1, 66.7%; dose level 2, 57.1%) and Part B (87.5%) were male. Most patients (58.3%) had stage IV disease. Patient demographics and disease characteristics at baseline are presented in Table 1 for this Phase 1 study population. As prior therapy, patients received radiotherapy (Part A, dose levels 1 and 2, *n* = 100%; Part B, *n* = 100%), systemic and locoregional therapy (Part A, dose level 1, *n* = 66.7%; Part A, dose level 2, *n* = 100%; Part B, *n* = 12.5%), and surgical procedure (Part A, dose level 1, *n* = 66.7%; Part A, dose level 2, *n* = 100%; Part B, *n* = 12.5%).

**TABLE 1:** Patient and disease characteristics of Japanese patients with advanced/metastatic cancers

Characteristics	Part A		Part B
	LY2801653 80 mg (*n* = 3)	LY2801653 120 mg (*n* = 7)	LY2801653‐80 mg/Cisplatin‐25 mg/m^2^ IV/Gemcitabine‐1000 mg/m^2^ IV (*n* = 8)
Age, years, median (range)	51 (43–60)	63 (43–73)	62.5 (47–72)
Female, %	33.3%	42.9%	12.5%
BMI, kg/m^2^, median (range)	23.301 (21.20–26.19)	20.320 (17.57–26.72)	21.715 (17.74–31.64)
ECOG PS, *n* (%) 0 1	2 (66.7) 1 (33.3)	4 (57.1) 3 (42.9)	7 (87.5) 1 (12.5)
Cancer type	Solid tumors	Solid tumors	Biliary tract cancer
Disease stage (study entry), *n* Stage IIIB Stage IV	0 3	0 7	2 6
Patients with prior systemic therapy, *n*	2	7	1
Patients with prior surgery, *n*	1	7	2
Patients with prior radiotherapy, *n*	2	4	0

Abbreviations: BMI, body mass index; ECOG, eastern cooperative oncology group; *n*, number of patients.

### 3.2 Safety

All patients in the study received merestinib; however, the relative dose intensity of merestinib across both parts of the study was different (Part A, dose level 1, *n* = 98.7%; Part A, dose level 2, *n* = 98.2%; Part B, *n* = 84.3%). Similarly, the cycles received per patient (patient is considered to have received a treatment cycle after receiving at least one dose of merestinib, gemcitabine, or cisplatin) also varied across Parts A and B (Part A, dose level 1, *n* = 3; Part A, dose level 2, *n* = 2; Part B, *n* = 7).

No DLT was reported in the DLT evaluable population. DLT equivalent toxicity was experienced by one patient (12.5%) in Part B. This patient experienced Grade 4 platelet count decreased on Study Day 210. The event was assessed as serious and related to the study treatment by investigator. The patient discontinued study treatment due to platelet count decreased on Study Day 225. No patients died during the study. A list of all AEs related to study treatment was classified according to maximum common terminology criteria for adverse events (CTCAE) Version 4 categories has been presented in Table 2 (Part A) and Table 3 (Part B).

**TABLE 2:** Summary of adverse events related to study treatment by maximum CTCAE grade categories (Part A)

Preferred term	Part A			
	LY2801653		LY2801653	
	80 mg (*n* = 3)		120 mg (*n* = 7)	
	Any grade	Grade ≥3	Any grade	Grade ≥3
Nausea	1	0	1	0
Decreased appetite	1	0	1	0
Alanine aminotransferase increased	2	0	4	1
Aspartate aminotransferase increased	2	0	4	1
Gamma‐glutamyl transferase increased	1	0	2	0
Constipation	1	0	0	0
Blood creatinine phosphokinase increased	0	0	2	0
Blood lactate dehydrogenase increased	0	0	1	0
Blood alkaline phosphatase increased	0	0	1	0
Edema peripheral	0	0	1	0

Abbreviations: AEs, adverse events; CTCAE version 4.

**TABLE 3:** Summary of adverse events related to study treatment by maximum CTCAE grade categories (Part B)

	Part B LY2801653‐80 mg/Cisplatin‐25 mg/m^2^ IV/Gemcitabine‐1000 mg/m^2^ IV (*n* = 8)	
Preferred term	Any grade	Grade ≥3
Nausea	3	0
Decreased appetite	3	0
Alanine aminotransferase increased	3	1
Aspartate aminotransferase increased	3	1
Gamma‐glutamyl transferase increased	1	0
Constipation	3	0
Blood alkaline phosphatase increased	1	0
Fatigue	2	0
Platelet count decreased	6	2
Alopecia	1	0
Pyrexia	4	0
Hemoglobin decreased	1	0
Blood creatinine increased	3	0
Leukopenia	1	0
Neutropenia	1	0
Neutrophil count decreased	6	4
White blood cell count decreased	5	1
Edema	1	0
Anemia	4	2
Neuropathy peripheral	1	0
Stomatitis	2	0
Rash	3	0
Vascular pain	1	0
Hiccups	1	0

In Part A dose level 1 (merestinib 80 mg), there was one serious adverse event (SAE) (Preferred Term [PT]: bile duct stenosis, Grade 3). This SAE was reported by the investigator to be not related to study treatment. The most frequently occurring TEAEs were alanine aminotransferase (ALT) increased, aspartate aminotransferase (AST) increased, and pyrexia (two patients each). One patient in Part A dose level 1 experienced Grade ≥3 TEAE (PT: bile duct stenosis, Grade 3) which was deemed not related to study treatment by the investigator. None of the patients in Part A dose level 1 discontinued study treatment due to AE.

In Part A dose level 2 (merestinib 120 mg), there was one SAE (PT: lung abscess, Grade 3). This SAE was reported by the investigator to be not related to study treatment. The most frequently occurring TEAEs were ALT increased and AST increased (57.1% each). Three patients experienced Grade ≥3 TEAEs: one patient had ALT increased and AST increased (related to study treatment), one patient had white blood cell count decreased, neutropenia, and lung abscess (none related to study treatment), and one patient had hypoalbuminemia (not related to study treatment). One patient discontinued the study treatment due to AE (PT: lung abscess). This event was not considered to be related to study treatment.

In Part B, there were six SAEs in five patients. Serious adverse events that were reported ≥2 patients included platelet count decreased (two patients). In two of the five patients with SAEs, there were two SAEs (platelet count decreased, *n* = 2) determined by investigator to be possibly related to study treatment. The most frequently occurring TEAEs were platelet count decreased (87.5%), pyrexia (87.5%), constipation (75.0%), anemia (75.0%), and neutrophil count decreased (75.0%). All patients in Part B had Grade ≥3 TEAE. Grade ≥3 TEAEs that were experienced by ≥50% of patients in Part B was neutrophil count decreased (4 [50.0%]). Three patients experienced Grade 4 TEAEs with all three patients reporting neutrophil count decreased and two of the three patients reporting platelet count decreased as well. In addition, all patients in Part B discontinued study treatment on account of three reasons: PD (*n* = 5; 62.5%), AEs (2; *n* = 25%), and withdrawal by subject (*n* = 1; 12.5%). AEs including platelet count decreased and ascites caused study treatment discontinuation, among which platelet count decreased was considered related to study treatment. Dose adjustments of merestinib for AEs had to be performed for patients in both Part A and Part B and of gemcitabine and cisplatin for AEs in Part B. In Part A, six patients underwent dose adjustment for merestinib while in Part B, seven patients underwent dose adjustment for merestinib. In addition, in Part B, all eight patients underwent dose adjustment for gemcitabine and cisplatin.

### 3.3 Pharmacokinetics

**FIGURE 2:**
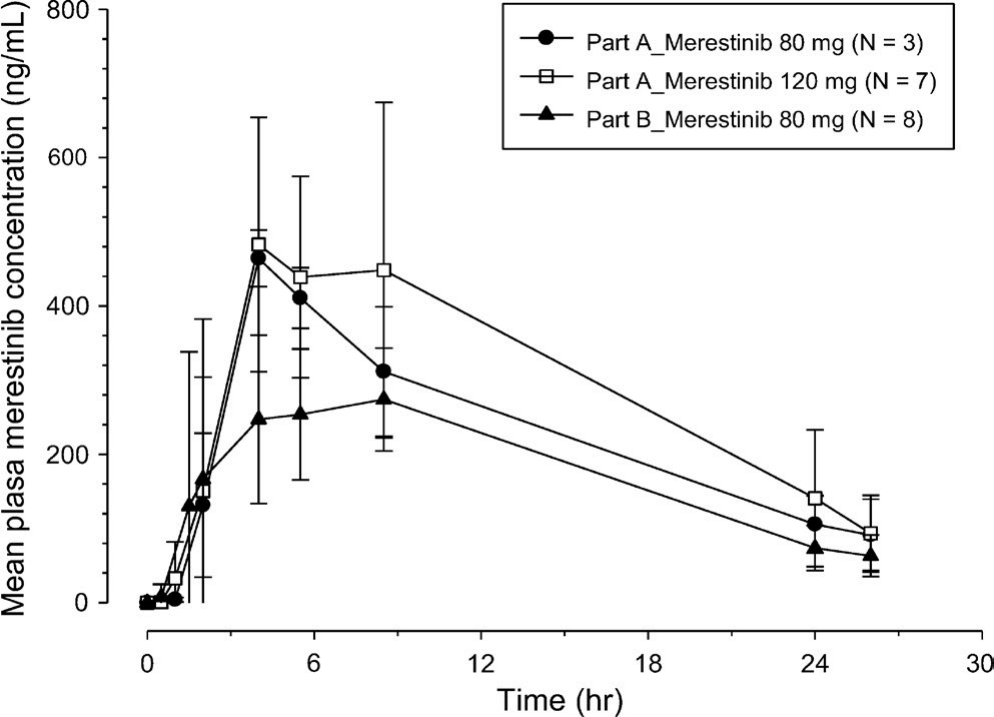
Arithmetic mean (±SD) plasma merestinib (LY2801653) concentration–time profiles on Day 1 in Cycle 1 in Japanese patients with advanced and/or metastatic cancer following once daily (QD) oral doses of 80 or 120 mg merestinib (Part A) and merestinib 80 mg in combination with cisplatin and gemcitabine (Part B)

**FIGURE 3:**
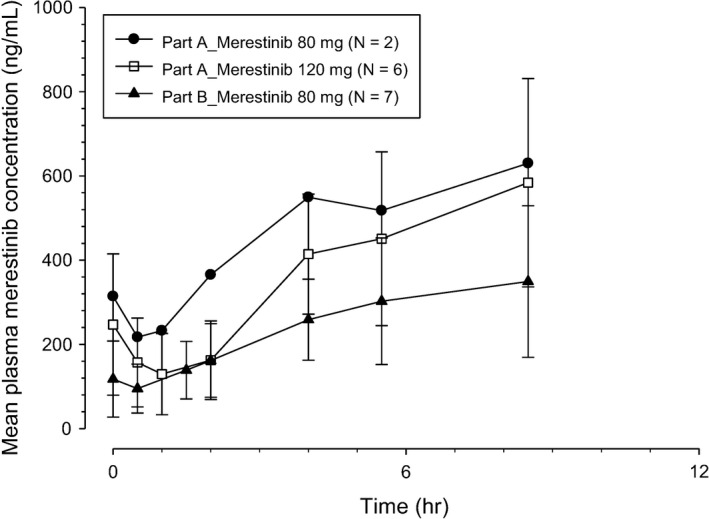
Arithmetic mean (±SD) plasma merestinib (LY2801653) concentration–time profiles on Day 1 in Cycle 2 in Japanese patients with advanced and/or metastatic cancer following once daily (QD) oral doses of 80 or 120 mg merestinib (Part A) and merestinib 80 mg in combination with cisplatin and gemcitabine (Part B)

All 18 enrolled patients who received ≥1 dose of the study treatment and had PK samples collected were included in the PK analyses. Figures 2 and 3 show mean plasma merestinib concentration–time profiles on Day 1 in Cycles 1 and 2, respectively, following once daily oral doses of merestinib 80 or 120 mg (Part A), and merestinib 80 mg in combination with cisplatin and gemcitabine (Part B). Table 4 summarizes corresponding PK parameters of merestinib on Day 1 in Cycles 1 and 2.

**TABLE 4:** Summary of merestinib pharmacokinetic parameters in Japanese patients with advanced and/or metastatic cancer following QD oral doses of 80 or 120 mg merestinib (Part A) and merestinib 80 mg in combination with cisplatin and gemcitabine (Part B)

Geometric mean (%CV)			
Cycle 1 Day 1 (last sampling time: 26 h post‐dose)			
	80 mg (Part A)	120 mg (Part A)	80 mg (Part B)
*N* ^a^	3/3	7/5	8/5
*t*_max_ ^b^ (h)	4 (3.95–5)	4 (2–7.45)	4.75 (2–7.27)
*C*_max_ (ng/ml)	464 (8)	571 (33)	323 (40)
AUC_0–24_ (ng * h/ml)	4980 (23)	6240 (36)	3790 (24)
AUC_0–tlast_ (ng * h/ml)	5170 (24)	6460 (37)	3920 (24)
AUC_0–∞_ (ng * h/ml)	6490 (34)	7190 (40)	4570 (35)1
*T*_1/2_ ^c^ (h)	10 (7.43–11.7)	8.92 (6.22–13)	7.45 (6.09–9.58)
CL/F (L/h)	12.3 (34)	16.7 (40)	17.5 (35)
*V*_z_/*F* (L)	178 (11)	215 (27)	188 (22)

Cycle 2 Day 1 (last sampling time: 7–10 h post‐dose)			
	80 mg (Part A)	120 mg (Part A)	80 mg (Part B)
*N* ^a^	2	6	7
*t*_max_ ^b^ (h)	3.88, 7.05^d^	6.07 (3.97–7.45)	7.08 (0.00–7.13)
*C*_max_ (ng/ml)	506.95, 919.16^d^	604 (37)	355 (55)
AUC_0–24_ (ng * h/ml)	2760, 3610^d^	2350 (28)	1530 (43)

Abbreviations: %CV, percent coefficient of variation; AUC_0–24_, area under the plasma concentration versus time curve from time from 0 to 24 h; AUC_0–∞_, area under the plasma concentration versus time curve from time zero to infinity; AUC_0–tlast_, area under the plasma concentration versus time curve from time zero to time tlast; CL/F, apparent clearance; *C*_max_, maximum observed plasma concentration; h, hours; *N*, number of patients; PK, pharmacokinetic; QD, once daily; *t*_1/2_, elimination half‐life; *t*_max_, time to reach *C*_max_; *V*_z_/*F*, apparent volume of distribution.

^a^ Two *N* values reported. First *N* value is for *t*_max_, *C*_max_, AUC_0–24_, and AUC_0–tlast_, while the second *N* value is for remaining parameters that are dependent on terminal phase of PK profile.

^b^ Median (range).

^c^ Geometric mean (range).

^d^ Individual value when *N* = 2.

On Day 1 in Cycles 1 and 2 (Figures 2 and 3), absorption of merestinib was slow, with median time to maximum plasma concentration (*t*_max_) occurring approximately 4–5 h post‐dose in each dose level. The geometric mean *t*_1/2_ was approximately 8–10 h. The variability in exposures across all doses, as assessed by percent coefficient of variation (%CV), ranged between 8% and 40%. When comparing exposures following oral doses of merestinib 80 mg in Part A and Part B, exposure levels in Part B were somewhat lower than those in Part A.

Plasma concentrations—time profiles of metabolites LSN2800870 (Figure S1) and LSN2887652 (Figure S2) were similar with merestinib. The geometric mean t_1/2_, however, was slightly longer than merestinib, and ranged from approximately 9 to 14 h while the variability in exposures across all doses was slightly higher than merestinib, ranging between 19% and 69% (Table 5).

**TABLE 5:** Summary of merestinib metabolites (LSN2800870 and LSN2887652) pharmacokinetic parameters in Japanese patients with advanced and/or metastatic cancer following QD oral doses of 80 or 120 mg merestinib (Part A) and merestinib 80 mg in combination with cisplatin and gemcitabine (Part B)

Geometric Mean (%CV)						
	LSN2800870			LSN2887652		
Cycle 1 Day 1 (last sampling time: 26 h post‐dose)						
	80 mg (Part A)	120 mg (Part A)	80 mg (Part B)	80 mg (Part A)	120 mg (Part A)	80 mg (Part B)
*N* ^a^	3/1	7/3	8/5	3/1	7/2	8/4
*t*_max_ ^b^ (h)	5.0 (4.0–5.0)	5.1 (3.9–7.5)	5.3 (2.0–7.27)	4.0 (3.9–5.0)	4.0 (2.0–7.5)	6.3 (2.0–7.2)
*C*_max_ (ng/ml)	47.4 (19)	75 (69)	33.5 (58)	131 (28)	176 (55)	93.8 (34)
AUC_0–24_ (ng * h/ml)	595 (22)	972 (52)	433 (48)	1650 (24)	2290 (47)	1200 (22)
AUC_0–tlast_ (ng * h/ml)	625 (23)	1020 (52)	451 (47)	1740 (25)	2400 (47)	1260 (22)
AUC_0–∞_ (ng * h/ml)	623^d^	1260 (57)	671 (51)	2220^d^	2400, 2820^d^	1840 (21)
*T*_1/2_ ^c^ (h)	8.93^d^	9.03 (8.46–10.1)	8.73 (6.48–12.3)	13.6^d^	9.0, 9.9^d^	11.1 (9.5–13.7)

Cycle 2 Day 1 (last sampling time: 7–10 h post‐dose)						
	80 mg (Part A)	120 mg (Part A)	80 mg (Part B)	80 mg (Part A)	120 mg (Part A)	80 mg (Part B)
*N*	2	6	7	2	6	7
*t*_max_ ^b^ (h)	3.88, 7.0^d^	7.0 (3.9–7.4)	7.0 (0.0–7.1)	5.0, 7.0^d^	6.0 (3.9–7.4)	5.0 (0.0–7.1)
*C*_max_ (ng/ml)	70.1, 123.6^d^	114 (31)	51 (51)	168.5, 300.8^d^	311 (56)	137 (51)
AUC_0–tlast_ (ng * h/ml)	380, 550^d^	493 (25)	232 (55)	950, 1190^d^	1250 (41)	612 (52)

Abbreviations: %CV, percent coefficient of variation; AUC_0–24_, area under the plasma concentration versus time curve from time 0 to 24 h; AUC_0–∞_, area under the plasma concentration versus time curve from time zero to infinity; AUC_0–tlast_, area under the plasma concentration versus time curve from time zero to time tlast; *C*_max_, maximum observed plasma concentration; h, hours; *N*, number of patients; QD, once daily; *t*_1/2_, elimination half‐life; *t*_max_, time to reach *C*_max_.

^a^ Two *N* values reported. First *N* value is for *t*_max_, *C*_max_, AUC_0–24_, and AUC_0–tlast_, while the second *N* value is for remaining parameters that are dependent on terminal phase of PK profile.

^b^ Median (range).

^c^ Geometric mean (range).

^d^ Individual value when *N* = 2.

### 3.4 Antitumor activity

All patients in Part A (*n* = 10) had at least one post‐baseline tumor scan for response assessment and were considered evaluable according to RECIST v1.1; however, of the eight patients in Part B, two patients were considered non‐evaluable. No complete response (CR) was reported in Parts A and B. Although no partial response (PR) was reported in Part A, one patient reported PR in part B. A best overall response of SD was reported in four patients (two patients from each of the two dose levels) of Part A, and four patients of Part B. The ORR observed in patients in Part A was negligible while in Part B, it was reported to be 12.5%.

## 4 DISCUSSION

In recent years, MET/HGF inhibition has emerged as a promising anticancer therapy option since aberrant MET/HGF activation, which occurs via a myriad of mechanisms and oncogenic receptor pathways, has largely been linked with several aggressive cancer phenotypes, increased metastasis, and poor prognosis.^20^ Based on the results of a previously conducted global Phase 1 study of merestinib in patients with advanced cancer in the USA,^18^ this study was conducted with the primary objective of evaluating the tolerability of merestinib monotherapy or in combination with gemcitabine and cisplatin in Japanese patients with advanced and/or metastatic cancer. The results support the dosing of merestinib as a single agent at 120 mg once daily or at 80 mg once daily in combination with cisplatin and gemcitabine in the specified demographic.

Although in the global study DLTs of reversible asymptomatic Grade 3 increases in LFTs were noted at the highest tested doses, including merestinib 120 mg, no DLTs were observed in this study in either Part A (merestinib 80/120 mg monotherapy) or Part B (combination of merestinib 80/120 mg, gemcitabine and cisplatin).

All patient experienced ≥1 TEAEs. In Part A at any dose level, most common TEAEs were ALT increased (Part A total: 60.0%, dose level 1: 66.7%, dose level 2: 57.1%) and AST increased (Part A total: 60.0%, dose level 1: 66.7%, dose level 2: 57.1%) while in Part B most common TEAEs were platelet count decreased and pyrexia.

Only four patients experienced Grade ≥3 TEAEs in Part A while all patients in Part B (*n* = 8) experienced Grade ≥3 TEAEs. ALT increased and AST increased were observed in the patients treated with merestinib alone in the global study, as treatment‐related Grade ≥3 TEAE. Similarly, in this study as well, one patient in Part A reported treatment‐related Grade ≥3 TEAE of ALT increased and AST increased. In addition, one patient in Part B reported treatment‐related Grade ≥3 TEAE of neutrophil count decreased, platelet count decreased, white blood cell count decreased, anemia, ALT increased, and AST increased, all of which except AST increased were observed in the patients treated with merestinib, gemcitabine, and cisplatin in the global study as treatment‐related Grade ≥3 TEAE. These findings indicate that the safety profiles of merestinib determined in both studies might be comparable. Furthermore, the increased occurrence of events of AST increased and ALT increased is also consistent with the results of other Phase 1 MET inhibition studies (NCT01553656, NCT01832506) in Japanese patients with tumors wherein more than 90% of patients had experienced both events of any grade.^21^, ^22^

The PK profile of merestinib was characterized by slow absorption and was generally consistent with PK data reported previously. In addition, similar to the global study, exposures (AUC_0–24_) after single doses of 80 and 120 mg reached or exceeded corresponding thresholds (that met the EC50 threshold for tumor growth inhibition) associated with efficacy in a preclinical (mouse) U87MG xenograft PK‐PD model.

Although the study was not designed for efficacy assessment, antitumor activity was evaluated as a secondary objective. No CR was observed while one patient in Part B achieved PR. Overall, in Parts A and B combined, 8 of 18 (44%) had a best response of SD which may be attributed to the versatility of merestinib in that in addition to primarily inhibiting MET, it also inhibits serine/threonine kinases MKNK1 and MKNK2 and receptor tyrosine kinases MST1R, TYR03, TEK, TRKA, AXL, FLT3, DDR1, TRKB, ROS1, PDGFRA, DDR2, MERTK, and TRKC.^16^ Furthermore, the ORR observed in patients of Part B (ORR = 12.5%) was similar to the ORR observed in patients who received merestinib in combination with cisplatin and gemcitabine (ORR = 18.8%) in the global study.

The study was limited by the non‐randomized study design and the small sample size, although these are typical characteristics of dose‐escalation studies. Since a key concern related to MET inhibition clinical trials is patient selection, it may be worthwhile to explore potential predictive biomarkers that may help identify patients most likely to benefit from MET inhibition therapy.^23^

## 5 CONCLUSIONS

Overall, based on the results of this Phase 1 study in Japanese patients with advanced/metastatic malignancies, merestinib monotherapy, and the combination of merestinib with cisplatin and gemcitabine appears to be tolerable regimens for Japanese patients. The safety profile was consistent with monotherapy for each drug, with no additive toxicities and no unexpected safety signals were seen to date.

## DISCLOSURE STATEMENT

**TD** received consulting fees or honorarium from Amgen, MSD, Taiho, Boehringer Ingelheim, Abbvie, Takeda, Rakuten Medical, Daiichi Sankyo; payment for lectures from Abbvie, Bristol Myers Squibb, Astellas, Ono Pharma, Oncolys BioPharma, Taiho, Rakuten Medical; and grants from Lilly, Taiho, Novartis, Merck Serono, MSD, Janssen, Boehringer Ingelheim, Eisai, IQVIA, Sumitomo Dainippon, Daiichi Sankyo, Bristol Myers Squibb, Abbvie. **NY** received honoraria from BMS, Pfizer, AstraZeneca, Eli Lilly, ONO, Chugai, Sysmex and research funds from Astellas, Chugai, Eisai, Taiho, BMS, Pfizer, Novartis, Eli Lilly, AbbVie, Daiichi‐Sankyo, Bayer, Boehringer Ingelheim, Kyowa‐Hakko Kirin, Takeda, ONO, Janssen Pharma, MSD, MERCK, GSK, Sumitomo Dainippon, Chiome Bioscience. **YN** received lecture fees from Chugai, Pfizer, and Novartis and research funds from Roche. **YK** received grants from Amgen, Takeda, AstraZeneca, Ono Pharmaceutical Company Limited, Taiho Pharmaceutical Company Limited, Boehringer Ingelheim GmbH. AbbVie, GSK, Chugai Company Limited, Daiichi‐Sankyo and Genmab K.K; served as a consultant at Takeda; received personal fees from Taiho, Sanofi, Bayer Yakuhin, and Ono Pharmaceutical Company Limited. **TK** received honoraria from Cysmex and Chugai. **YP**, **KI**, **NT,** and **HA** are employees of Eli Lilly Japan K.K. **SK** received research funding from ASLAN Pharmaceuticals, AstraZeneca, Bayer, Eli Lilly, MSD, Boehringer Ingelheim, and Pfizer. The study was designed under the responsibility of Eli Lilly and Company, in conjunction with the steering committee; The study was funded by Eli Lilly and Company. Merestinib was provided by Eli Lilly and Company. Eli Lilly and Company collected and analyzed the data and contributed to the interpretation of the study. All authors had full access to all of the data in the study and had final responsibility for the decision to submit for publication.

## ETHICAL APPROVAL

The study and all procedures performed in the study involving human participants were conducted in accordance with ethics principles derived from international ethics guidelines, including the Declaration of Helsinki and the Council for International Organizations of Medical Sciences International Ethical Guideline and the International Conference on Harmonization E6 Guidelines for Good Clinical Practice.

## CONSENT TO PARTICIPATE

Informed consent was obtained from all individual participants included in the study.

## Supporting information

Fig S1A

Fig S1B

Fig S2A

Fig S2B

## Data Availability

Eli Lilly and Company provides access to all individual participant data collected during the trial, after anonymization, with the exception of pharmacokinetic or genetic data. Data are available to request 6 months after the indication studied has been approved in the United States and European Union and after primary publication acceptance, whichever is later. No expiration date of data requests is currently set once data are made available. Access is provided after a proposal has been approved by an independent review committee identified for this purpose and after receipt of a signed data sharing agreement. Data and documents, including the study protocol, statistical analysis plan, clinical study report, and blank or annotated case report forms, will be provided in a secure data‐sharing environment. For details on submitting a request, see the instructions provided at www.vivli.org.
